# Immune-Related Esophagitis and Durable Response to Second-Line Treatment With Nivolumab in Stage IV Squamous Cell Esophageal Carcinoma

**DOI:** 10.7759/cureus.98324

**Published:** 2025-12-02

**Authors:** Olga Meneses, Maria Alzamora, Carla Dias, Raquel Ortigão, Dânia Marques

**Affiliations:** 1 Medical Oncology, Portuguese Institute of Oncology Francisco Gentil, Porto, PRT; 2 Pathology, Portuguese Institute of Oncology Francisco Gentil, Porto, PRT; 3 Gastroenterology, Portuguese Institute of Oncology Francisco Gentil, Porto, PRT

**Keywords:** complete response, esophageal squamous cell carcinoma, esophagitis, immune-related adverse effects, nivolumab

## Abstract

Immune checkpoint inhibitors such as nivolumab have transformed the management of advanced cancers but may lead to immune-related adverse events, including rare toxicities affecting the upper gastrointestinal tract. Isolated immune-mediated esophagitis is uncommon, particularly in esophageal squamous cell carcinoma, and its clinical implications remain poorly characterized.

We report the case of a 78-year-old male with advanced esophageal squamous cell carcinoma who developed epigastric pain, dysphagia, and weight loss seven months after initiating nivolumab therapy. Endoscopy revealed ulcerative esophagitis, and histology confirmed chronic inflammation, with infectious etiologies ruled out. Nivolumab was discontinued, and the patient was managed with proton pump inhibitors and high-dose corticosteroids, resulting in significant symptom improvement. At the time of immunotherapy discontinuation, the patient had achieved a radiologic partial response. Remarkably, during follow-up without further oncologic therapy, his disease continued to regress, culminating in a complete response that has been sustained for twenty-one months.

This case is notable for the unique combination of advanced esophageal squamous cell carcinoma, isolated nivolumab-induced esophagitis, and a durable tumor response sustained long after immunotherapy discontinuation, underscoring the importance of recognizing immune-mediated toxicities and their potential association with robust antitumor activity.

## Introduction

Esophageal cancer represents a major global health concern. In 2020, more than 600,000 cases and 540,000 deaths were reported worldwide [1,2]. Esophageal squamous cell carcinoma (ESCC) constitutes the predominant histological subtype. In response to this disease burden, immune checkpoint inhibitors (ICIs) have emerged as key therapeutic options. Reflecting these advancements, current European Society of Medical Oncology guidelines recommend immunotherapy regimens for advanced ESCC, including nivolumab as monotherapy or in combination with chemotherapy, or ipilimumab and pembrolizumab for programmed death-ligand 1 (PD-L1)-positive tumors in selected patients. These recommendations apply to both first- and second-line settings, depending on PD-L1 expression [3].

Nivolumab is a fully human IgG4 monoclonal antibody that targets programmed cell death protein 1 (PD-1), thereby restoring anti-tumor immunity by blocking PD-1/programmed death-ligand 1 (PD-L1) interactions and reactivating T-cell responses. Its efficacy in previously treated advanced ESCC is supported by the phase 3 ATTRACTION-3 trial, which demonstrated improved overall survival compared with chemotherapy (median OS 10.9 vs 8.4 months; HR 0.77), although progression-free survival was shorter (1.7 vs 3.4 months) [4].

Despite their efficacy, ICIs can cause immune-related adverse events (irAEs), resulting from heightened immune activation. The development of irAEs is associated with survival benefits in melanoma [5], non-small cell lung cancer [6], and renal cell carcinoma [7]. While gastrointestinal irAEs are recognized, upper gastrointestinal tract involvement is rare and is less well characterized than colitis [8]. For this reason, a careful diagnosis is essential. Esophagitis refers to inflammation of the esophageal mucosa, typically caused by infectious, medication-related, radiation-induced, reflux-associated or immune-mediated processes, and the diagnosis of immune-mediated esophagitis requires endoscopic evaluation and histological assessment [9]. Small retrospective series suggest a possible association between irAEs and improved outcomes in upper gastrointestinal cancers, including ESCC [10,11]. Durable tumor control after ICI discontinuation has also been reported, although available evidence remains limited [12].

These findings highlight a critical clinical challenge: managing severe irAEs without compromising therapeutic benefit. We present what we believe to be the first case of ESCC who discontinued nivolumab due to immune-related esophagitis, maintaining a durable response for 21 months after stopping nivolumab.

## Case presentation

A 78-year-old male was diagnosed with unresectable squamous cell carcinoma of the middle esophagus. The cancer was classified as stage cT4N+M0 and showed a mismatch repair proficiency phenotype. Tumor mutational burden, determined by next-generation sequencing, was within normal limits. The patient reported moderate alcohol intake, estimated at approximately 36 grams of ethanol per day. He denied any history of tobacco use. His modified Charlson index was three, and his ECOG performance status was one.

Initial treatment began in June 2020 with radical chemoradiotherapy, continuing until September 2020, and resulted in a partial response as the best overall outcome. Fifteen months later, unresectable pulmonary recurrence was observed. First-line palliative therapy with cisplatin (75 mg/m² on day one) and 5-fluorouracil (750 mg/m²/day as a continuous infusion on days 1-4), administered every 3 weeks, resulted in stable disease as the best response. After six cycles, disease progression was noted, including enlargement of a right upper lobe pulmonary metastasis, emergence of new pulmonary metastatic nodules, and increased size of multiple mediastinal and peritumoral lymphadenopathies, as well as progression of the primary esophageal tumor. Nivolumab therapy was initiated at a dose of 480 mg every 28 days in May 2023. During this period, the patient developed grade 1 hypothyroidism, which was treated with levothyroxine replacement therapy. Comparison between baseline imaging and the October 2023 scan demonstrated a radiologic partial response.

**Figure 1:**
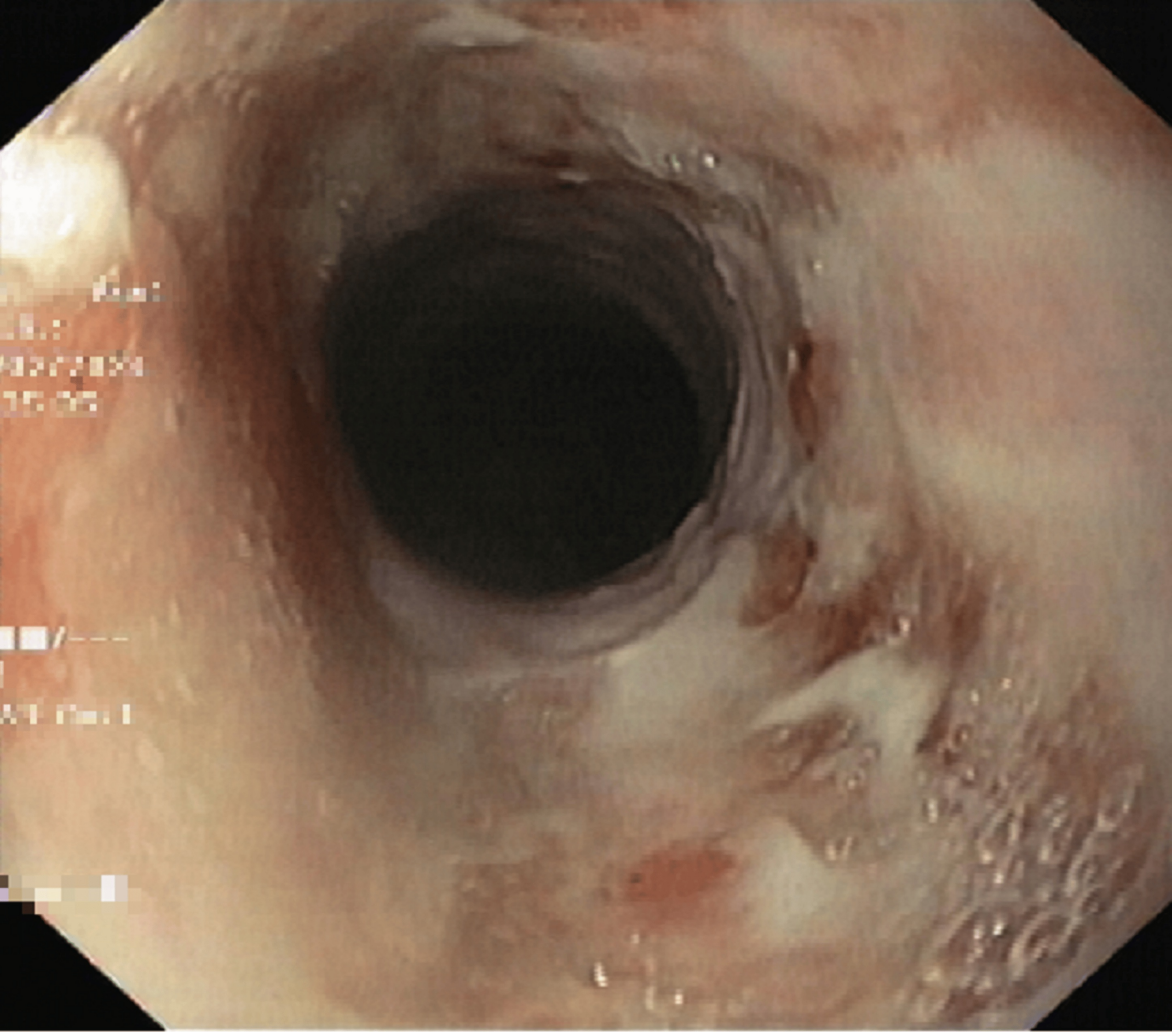
Endoscopic view of the esophagus showing areas of mucosal irregularity, ulcerations, and whitish exudate suggestive of inflammatory involvement.

**Figure 2:**
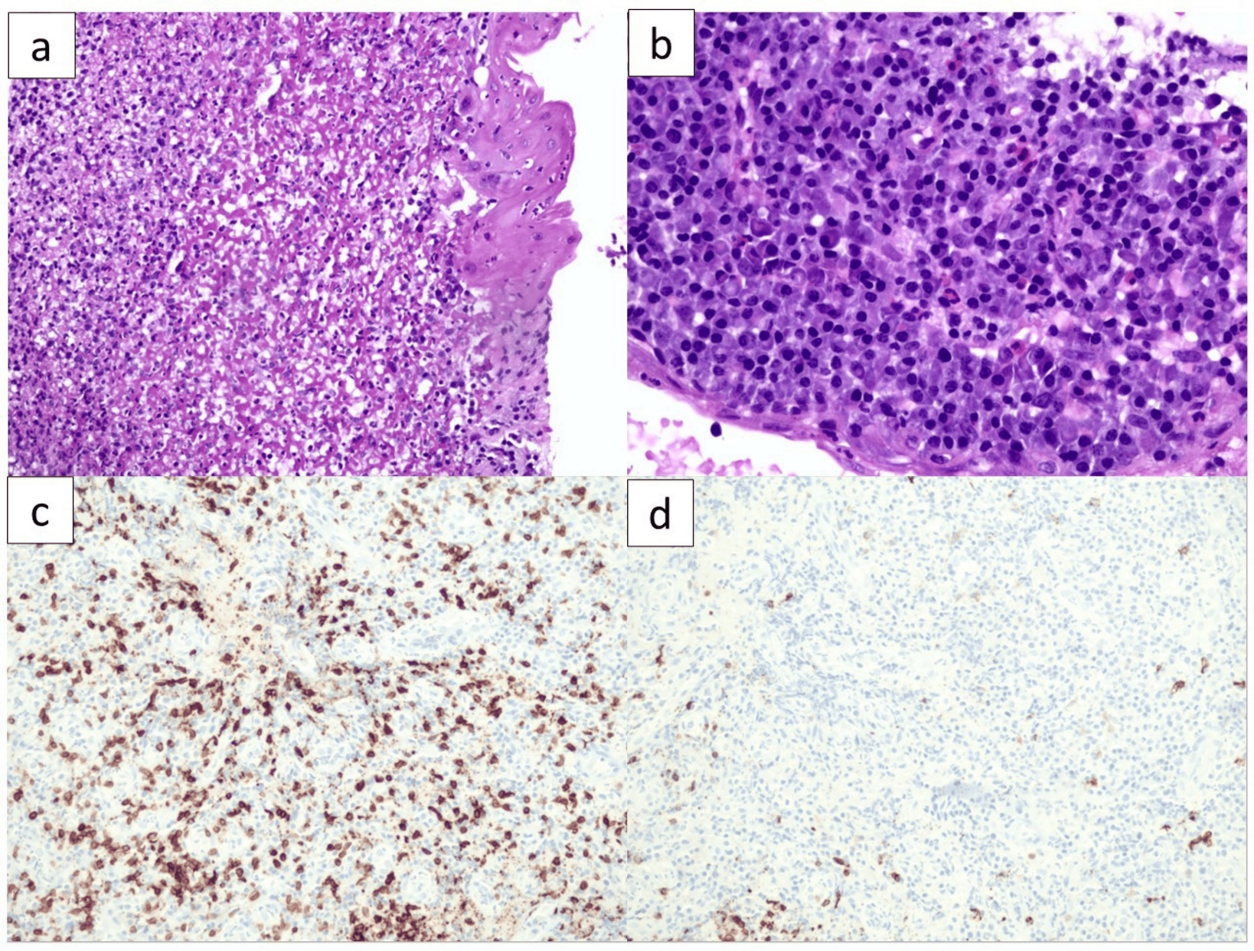
Esophageal biopsy specimen. (A) Hematoxylin and eosin (H&E)-stained section at 100x magnification, demonstrating esophageal ulceration with intraepithelial lymphocytosis and underlying granulation tissue. (B) H&E-stained section at 400x magnification, highlighting chronic active inflammation. (C and D) CD3 and CD20 immunohistochemistry at 200x magnification, showing a predominance of CD3+ T-lymphocytes in the inflammatory infiltrate.

In December 2023, he presented to the emergency department of our institution with a one-week history of progressive dysphagia, moderate epigastric pain unrelated to food intake, and episodes of vomiting shortly after food intake, along with a 5% unintentional weight loss over the previous month. Initial laboratory evaluation confirmed that thyroid function and cortisol levels remained within normal ranges, ruling out endocrine abnormalities as potential contributors. To further investigate the etiology of his symptoms, an upper endoscopy was performed (Figure 1), and biopsy specimens were obtained (Figure 2). No colonoscopy was carried out due to the absence of symptoms in the lower gastrointestinal tract. Microbiological testing for herpes simplex virus, cytomegalovirus, fungi, and Helicobacter pylori was negative.

A presumptive diagnosis of grade 2 esophagitis secondary to nivolumab was made. Nivolumab was discontinued, and the patient was started on a proton pump inhibitor (PPI) twice daily and antiemetics, but no clinical improvement was observed after three weeks. He was then initiated on prednisolone at 1 mg/kg/day for three weeks, with concurrent prophylaxis for opportunistic infections as well as vitamin D and calcium supplementation. The patient experienced marked improvement in symptoms, was able to tolerate a soft-consistency diet again, regained weight, and resumed daily activities. Prednisolone was subsequently tapered gradually over six weeks down to 10 mg/day. Histological evaluation of the biopsy obtained during endoscopy revealed ulcerative esophagitis with underlying chronic active inflammation.

**Figure 3:**
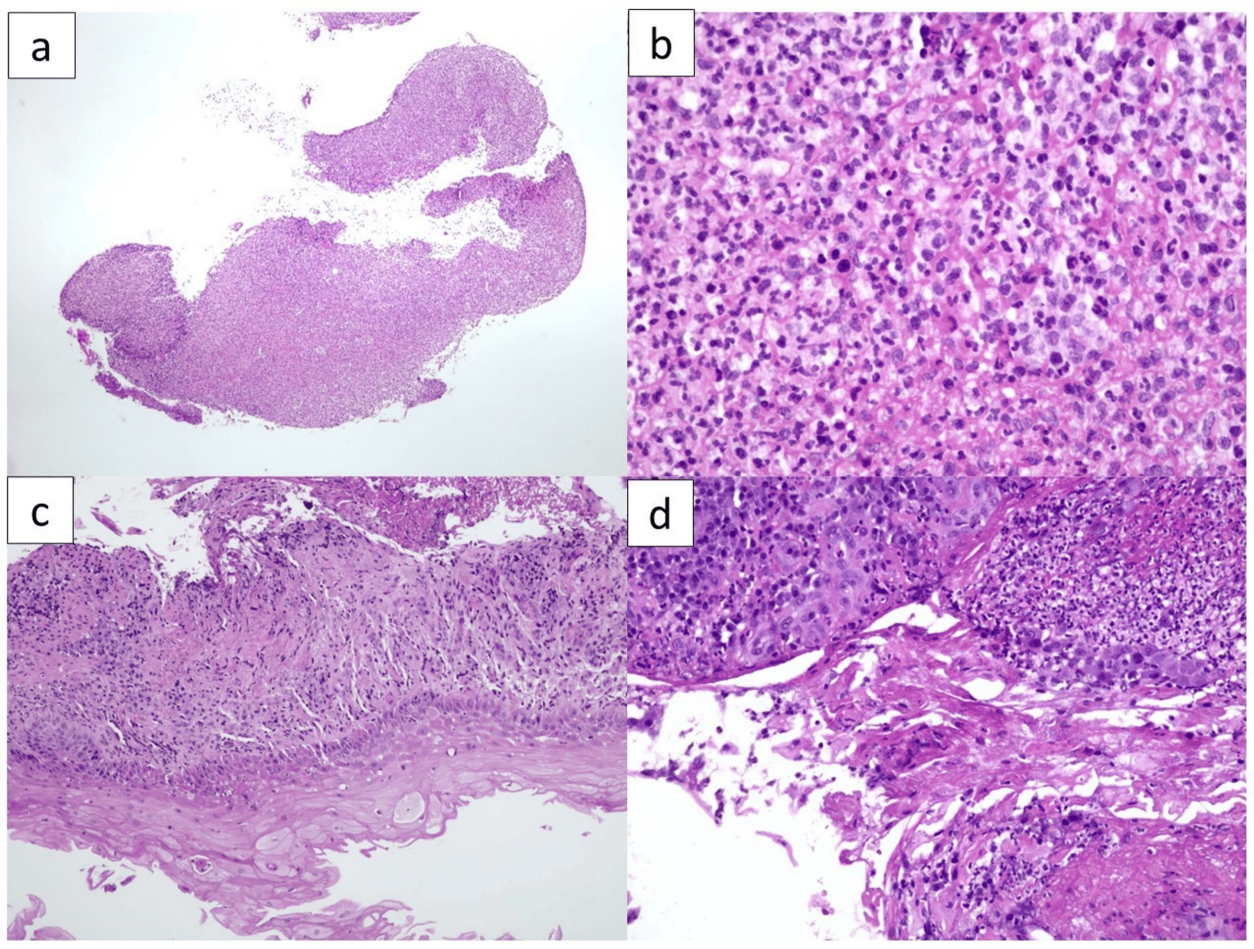
Esophageal biopsies at two- and five-month follow-up. (A and B) Hematoxylin and eosin (H&E)-stained sections at 40x and 400x magnification, demonstrating persistent ulcerative esophagitis with ongoing activity. (C and D) H&E-stained sections at 100x and 200x magnification, showing continued ulceration along with reparative epithelial features, without evidence of malignant disease recurrence.

Two and five months later, follow-up endoscopies were performed (Figure 3), showing persistent grade 1 esophagitis without the presence of neoplastic cells. Subsequent computed tomography assessments demonstrated sustained complete response.

**Figure 4:**
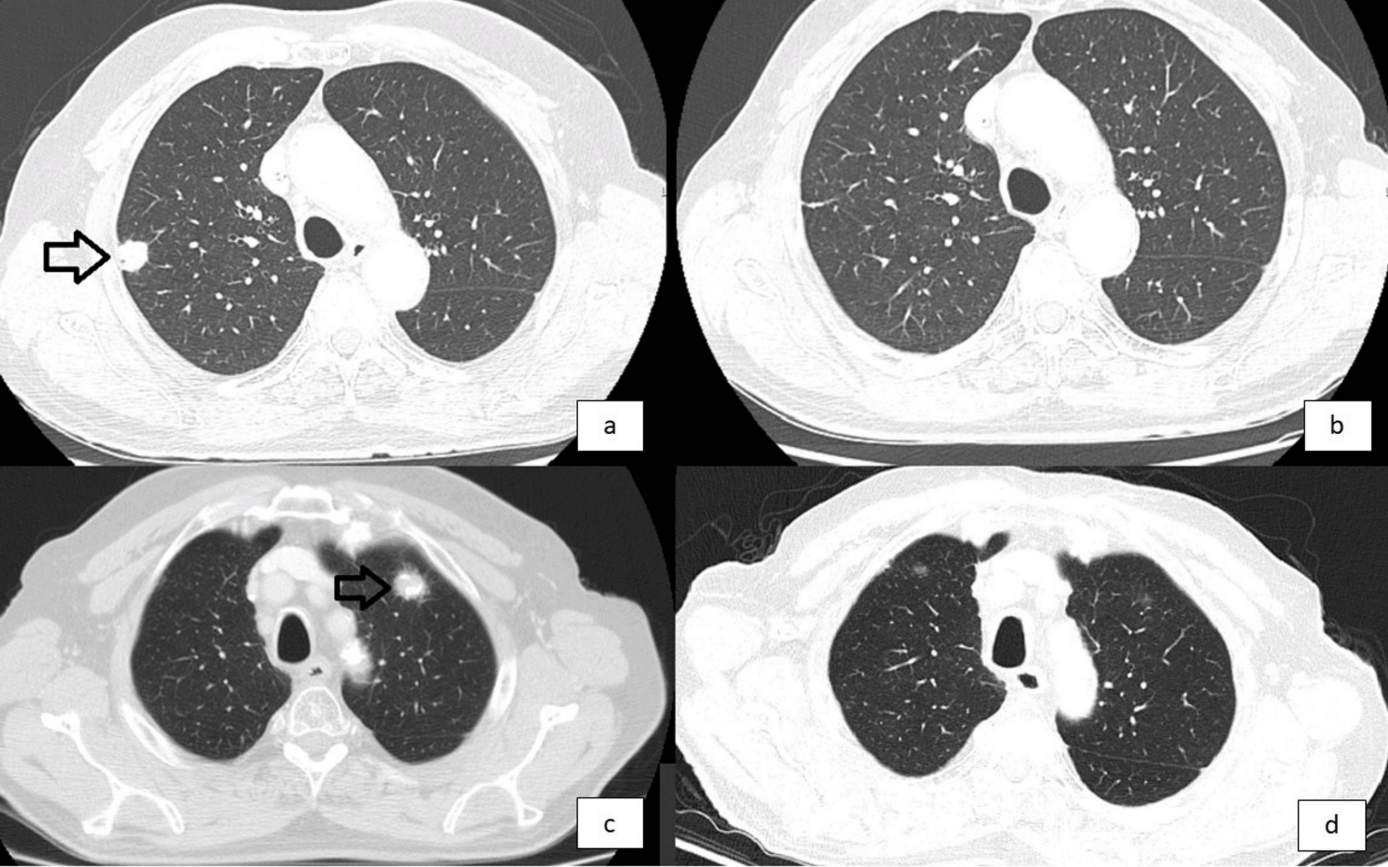
Comparison of pulmonary metastases before (May 2023) and after (September 2025) discontinuation of nivolumab therapy Panels A and C: Axial chest CT images demonstrating right upper lobe pulmonary metastases (arrows) before initiation of nivolumab. Panels B and D: Corresponding follow-up images showing a complete radiologic resolution of the metastatic lesions twenty-one months after suspension of nivolumab.

The patient remains clinically asymptomatic and fully independent in all basic and instrumental activities of daily living. At the most recent evaluation in September 2025, no measurable lesions were identified, consistent with a radiologic complete response (Figure 4). He has now maintained an immunotherapy-free interval of 21 months.

## Discussion

This report describes a patient with advanced ESCC who achieved a sustained tumor response to nivolumab despite discontinuation of treatment following biopsy-confirmed immune-related esophagitis (irAE). Adverse events associated with PD-1 inhibitors arise from disruption of immune self-regulatory pathways and may manifest as autoimmune-like toxicities [8]. In the ATTRACTION-3 trial, 66.3% of patients receiving nivolumab experienced at least one adverse event, 18.2% experienced grade 3-4 toxicity, and 8.6% discontinued treatment due to adverse events [4]. Gastrointestinal irAEs occur in up to 35% of cases [13], although esophageal involvement is notably uncommon. In the retrospective analysis by Panneerselvam et al. [13], only 3% of patients developed esophagitis, suggesting that the squamous esophageal mucosa may be less susceptible to PD-1-mediated injury than the columnar epithelium of other gastrointestinal segments [14]. When upper gastrointestinal irAEs occur, enterocolitis may coexist [13]. In our patient, the presentation was limited to isolated esophagitis, an entity reported in fewer than 15% of PD-1 inhibitor-related esophageal toxicities [13]. Symptoms first developed seven months after starting nivolumab, consistent with the typical onset window for upper gastrointestinal irAEs, which generally arise four to nine months after treatment initiation [13].

The diagnosis of immune-mediated esophagitis is one of exclusion. Upper endoscopy with biopsies is essential to exclude infectious etiologies, such as herpes simplex virus, cytomegalovirus, fungal pathogens, and Helicobacter pylori. Endoscopic findings may include erythema, ulcerations, edema, or stenosis, while histopathology commonly reveals lymphocytic infiltration or ulcerative changes [14]. In this patient, the differential diagnosis also included tumor recurrence and inflammatory changes related to prior chemoradiation. Although these possibilities could not be entirely excluded, the clinical evolution, biopsy findings, and temporal relationship with immunotherapy strongly supported an irAE.

Because endoscopic severity does not always correlate with symptoms, treatment is primarily based on clinical presentation [9,13,15]. Most mild cases improve with proton pump inhibitors or H2 blockers, usually within 30 days [13]. For more severe or persistent cases, treatment requires stopping immunotherapy and starting high-dose corticosteroids (approximately 1 mg/kg/day) [14]. Additional immunosuppressive agents such as anti-IL-6 therapy (tocilizumab) may be considered for steroid-resistant cases [16]. In the present case, symptoms improved significantly after three weeks of corticosteroids, with no need for further immunosuppression.

This case demonstrates that nivolumab monotherapy can result in a durable antitumor response in ESCC, even after discontinuation for a significant irAE. A key finding is the rare occurrence of isolated esophagitis as a PD-1 inhibitor-related toxicity with prolonged benefit from therapy. Reports suggest an association between irAEs and improved antitumor efficacy, helping explain the sustained response observed [5-7]. Notably, in our patient, the onset of esophagitis at seven months coincided with the period when a durable oncologic response became radiologically evident, supporting the hypothesis that irAEs may serve as a clinical marker of particularly effective antitumor immune activation. This is, to our knowledge, the first reported case of advanced ESCC showing prolonged tumor control following isolated esophagitis from a PD-1 inhibitor. If the disease progresses, rechallenging with nivolumab is an option, but careful consideration of risks and alternatives is essential. Multidisciplinary management informed both diagnosis and treatment decisions.

## Conclusions

This case illustrates a durable response to nivolumab in stage IV ESCC, sustained even after treatment discontinuation due to immune-related esophagitis. Complete symptom resolution with corticosteroid therapy, without the need for additional immunosuppressive agents, underscores the importance of vigilant monitoring and a multidisciplinary approach to irAEs. Notably, growing evidence suggests that antitumor activity may correlate with the occurrence and severity of irAEs, which may help explain why some patients, such as in this case, maintain prolonged clinical benefit even after immunotherapy withdrawal. Beyond highlighting the rarity of isolated esophagitis, this report emphasizes that long-lasting clinical benefit from immunotherapy may persist despite drug withdrawal, reinforcing the promising role of nivolumab in the management of advanced esophageal cancer.
